# Wide Skis As a Potential Knee Injury Risk Factor in Alpine Skiing

**DOI:** 10.3389/fspor.2020.00007

**Published:** 2020-02-18

**Authors:** Martin Zorko, Bojan Nemec, Zlatko Matjačić, Andrej Olenšek, Katja Tomazin, Matej Supej

**Affiliations:** ^1^Clinical Institute of Occupational, Traffic and Sports Medicine, University Medical Centre Ljubljana, Ljubljana, Slovenia; ^2^Department for Automatics, Biocybernetics and Robotics, Jožef Štefan Institute, Ljubljana, Slovenia; ^3^Research and Development Unit, University Rehabilitation Institute, Ljubljana, Slovenia; ^4^Faculty of Sport, University of Ljubljana, Ljubljana, Slovenia

**Keywords:** 3D kinematics, simulation, biomechanics, electromyography, injury prevention, kinetics, ski geometry

## Abstract

Alpine skis with wider waist widths have recently become more popular. With such skis, the contact point of the ground reaction force during ski turns is displaced more medially from beneath the sole of the outer ski, which may present an increased risk of injury. The aim of this study was to investigate knee joint kinetics, kinematics, and lower limb muscle activation as a function of changes of the ski waist width in a laboratory setting. A custom skiing simulator was constructed to enable simulation of different ski waist widths in a quasi-static ski turn position. An optical system was used for capturing knee joint kinematics of the outer leg, whereas a force plate was used to determine the ground reaction force vector. The combination of both systems enabled values for external torques acting on the knee joint to be calculated, whereas electromyographic measurements enabled an analysis of knee flexor muscle activation. With respect to the outer ski, the knee joint external torques were independent of ski waist width, whereas knee joint external rotation and biceps femoris activation increased significantly with the increase of the ski waist width. Skier muscle and kinematics adaptation most probably took place to diminish the external knee joint torque changes when the waist width of the ski was increased. The laboratory results suggest that using skis with large waist widths on hard, frozen surfaces may change the load of knee joint surfaces. However, future research is needed to clarify if this may result in the increased risk of knee injury.

## Introduction

Alpine skiing is a demanding sensory motor task with respect to maintaining balance and counteracting high external forces acting on the human body. It is well-documented that recreational alpine skiing is associated with high injury rates ranging from 2.4 to 7.0 injuries per 1,000 activity days (Hebert-Losier and Holmberg, 2013). Such injury rates are several times higher in competitive skiing compared with recreational skiing (Haaland et al., 2016). In addition to frequent acute injuries, overuse injuries are also common (Hildebrandt and Raschner, 2013; Sporri et al., 2015, 2016; Supej et al., 2017). The knee joint is the most frequently injured body part in both recreational and competitive skiing (Brucker et al., 2014; Haaland et al., 2016). More specifically, the outer knee in the ski turn has been reported to be more prone to acute injury compared with the inner one (Urabe et al., 2002). The relationships between sudden unexpected events and/or loss of balance accompanied by undesirable lower limb/knee movements while skiing and acute knee injuries have been well-established (Bere et al., 2011; Shea et al., 2014). However, the influence of ski equipment on chronic degenerative knee joint conditions is not yet clear.

Human gait analysis studies have proven that even small changes in knee joint alignment in the frontal plane, such as varus or valgus deformity, can substantially change the loading of different joint compartments and hasten degenerative processes of the joint (Sharma et al., 2001; Levine and Bosco, 2007). In addition, it has been shown that the human body tends to adapt to changes in body segment malalignment and asymmetrical loading of knee joint compartments (Mündermann et al., 2005). Nowadays, skis with very wide waist width on frozen compact snow are commonly used, although they were initially designed for “soft-snow” conditions, that is, powder and/or off-piste skiing. A previous study demonstrated that for ski turns on a hard snow base, an increase in ski waist width triggers adaptive changes of the knee kinematics in frontal and transversal planes (Zorko et al., 2015). This field study showed that knee external rotation increased with wider skis and that the knee joint was in valgus/abducted position throughout the ski turn, but, somewhat controversially, the valgus was smaller with wider skis. The main limitation of the study was that the degree of knee joint flexion while turning was uncontrolled and differed substantially between volunteers. As knee joint accessory movements (rotation and valgus/varus) are coupled with the primary movement of knee in the sagittal plane (flexion/extension) to some degree, the influence of ski waist width on the knee was somewhat unclear and uncertain.

**Figure 1:**
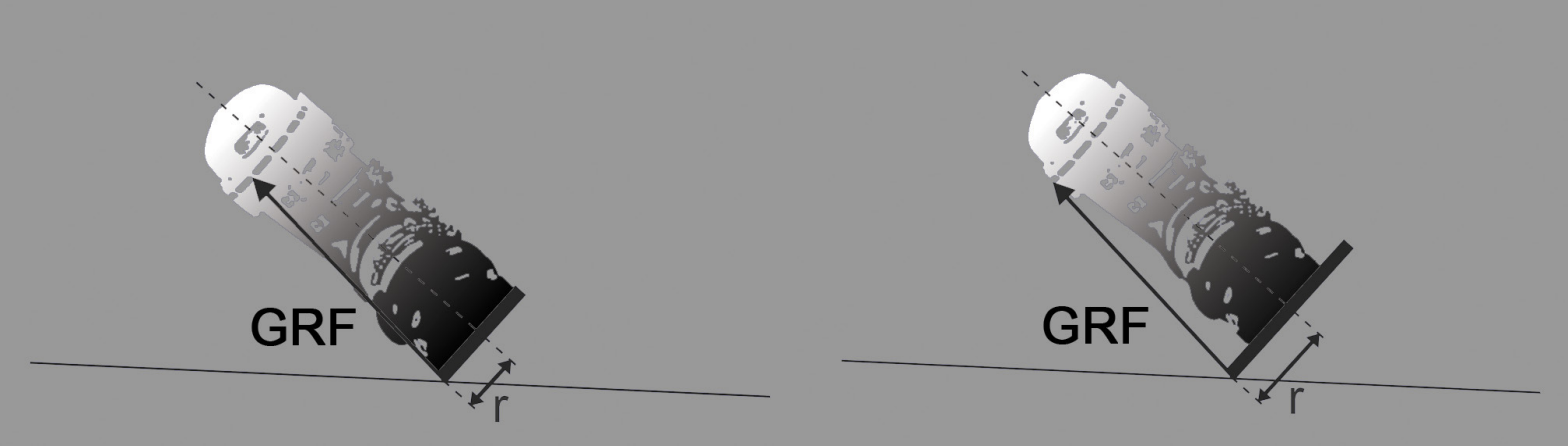
In a ski turn on hard frozen snow, the contact point of the ground reaction force (GRF) of the outside ski is shifted more medially with a wide ski (right: dw) than with a narrow one (left: dn).

With respect to the outer and more loaded ski involved in the turn (Vaverka and Vodickova, 2010), the point of application of the ground reaction force (GRF) moves from beneath the foot in the medial direction toward the ski edge (Figure 1). The greater the ski waist width, the greater the shift of GRF will be, assuming hard snow conditions where the ski does not sink into the snow (Federolf et al., 2010). Without changing knee kinematics, it can also be assumed that the alignment of GRF with respect to the center of the joint changes with the different ski waist width and accordingly also its torque. This hypothetical shifting of the GRF with respect to the center of the joint in relation to the separate knee compartment may represent a critical overload, because the magnitude of GRF reaches 1.5 to 2 times the body weight, even in recreational skiing (Scheiber et al., 2012), and up to four times the body weight in competitive skiing (Supej et al., 2004). Although the relationship between knee joint kinematics and the width of the skis during ski turns has already been demonstrated (Zorko et al., 2015), the influence of ski waist width on knee loading remains uninvestigated.

Therefore, the aim of the present study was to examine the knee joint kinetics, kinematics, and lower limb muscle activation with respect to differences in ski waist width. It was hypothesized that the wider waist width of the ski has a significant impact on knee position, external knee torque, and muscle activation. The experiment was conducted in a laboratory setting with the purpose of establishing more controlled conditions where vibrations (Klous et al., 2010) and other influences of uneven terrain (Federolf et al., 2009; Supej, 2013) are excluded.

## Materials and Methods

### Measurement System

**Figure 2:**
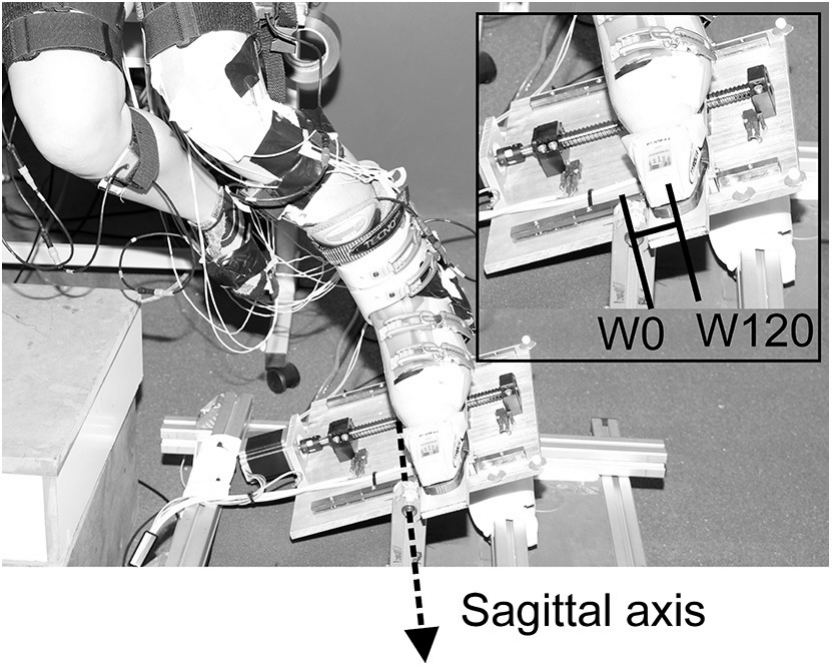
Skiing simulator placed on the force plate. The platform of the simulator was able to freely rotate around the sagittal axis. Two extremes of the four possible ski width positions are represented (W0 represents the position of the sagittal axis beneath the middle of the sole, and W120 represents the simulation of waist width of 120 mm).

**Figure 3:**
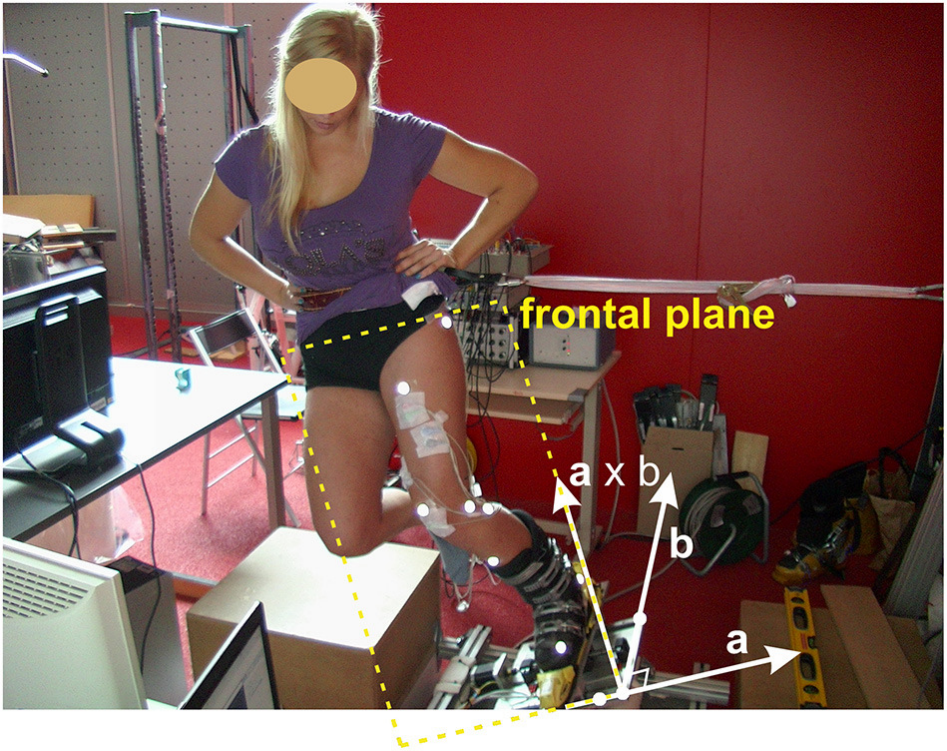
Participant bounded to ski turn simulator. The moving platform, optical markers, and the lateral supporting strap are seen. The frontal plane was calculated using three markers placed on the moving platform that defined two unit vectors. The first vector a was perpendicular, and the second vector b was parallel to the simulator's axis of rotation. One coordinate of the frontal plane was defined by vector b, and the other by the vector perpendicular to a and b (cross product of vectors a × b).

For the purpose of the study, a specially designed computer-controlled test device was built, enabling simulation of different ski waist widths for an outer leg in the quasi-static position of a ski turn (Figure 2). The test device comprised a movable plate equipped with ski bindings that allowed rotation around the sagittal axis in order to mimic the behavior of the outer ski. The inclination angle was measured with an inbuilt absolute magnetic encoder (RLS Renishaw RM22, Ljubljana, Slovenia). The test device was placed on a force plate (Kistler 9253A11, Kistler Group, Winterthur, Switzerland) measuring GRF vector and its point of application. Radial force (*F*_r_) was simulated and measured using a special harness attached to a load cell (S9M/2 kN, HBM GmbH, Darmstadt, Germany). Eleven reflective markers were placed in the following way: six on the outer leg, two on the ski boot, and three on the movable plate of the simulator (Figure 3); and they were captured by a system of three calibrated infrared cameras (Optitrack V120:Trio, Natural Point, USA).

During quasi-static skiing positions on the simulator, and during maximum isometric contractions that were used for subsequent normalization, surface electromyography (EMG) recordings were taken from the biceps femoris (BF) and semitendinosus (ST) muscles by a pair (2.5-cm inter-electrode distance) of bipolar self-adhesive electrodes (Ag-AgCl, type H124SG, Kendall). The electrodes were placed along the presumed direction of the underlying muscle fibers according to recommendations by SENIAM (Hermens et al., 2000) with the reference electrode placed over the patella. The electrodes were placed over the muscle belly. Electrodes for BF were placed at 50% on the line between the ischial tuberosity and the lateral epicondyle of the tibia. For the ST muscle, electrodes were placed at 50% on the line between the ischial tuberosity and the medial epicondyle of the tibia. Additionally, manual muscle testing was performed to ensure appropriate electrode placement. Before the electrodes were placed, the skin was shaved, cleaned, and abraded in order to optimize electrode–skin contact and gain low impedance (<5 kΩ) between electrodes. EMG data were recorded with a PowerLab system (16/30-ML880/P, ADInstruments, Bella Vista, Australia) at 2,000-Hz sampling frequency, amplified by octal bio-amplifiers (ML138, ADInstruments) with a bandwidth frequency ranging from 3 to 1,000 Hz (input impedance = 200 MΩ, common-mode rejection ratio = 85 dB, gain = 1,000), and analyzed using LabChart6 software (ADInstruments).

### Participants and Measurement Protocol

Fourteen healthy participants (8 males and 6 females; age: 27.9 ± 5.4 years; height: 174 ± 9 cm; weight: 73.9 ± 9.8 kg) with intermediate skiing skills participated in the study. Reflective markers and EMG electrodes were applied to the subjects (as described in *Measurement System*) prior to measurement. Initial measurements for surface EMG of the selected muscles during maximal isometric knee extension [maximal voluntary contraction (MVC)] were taken. For isometric measurements, participants sat in an isometric torque measuring device (own design) equipped with a strain gauge force sensor (MES, Maribor, Slovenia) set at the constant lever arm to the rotational axis of the knee. Subjects were seated in an isometric knee torque measuring device with left knee at 60° and 40° (0° full extension), respectively, for knee extensor and flexor MVC assessment, and hips were at 110° for each. The lower leg was attached to the isometric frame above the ankle joint (i.e., lateral malleolus). The rotational axis of the frame was visually aligned to the rotational axis of the knee (i.e., lateral femoral epicondyle) for each subject prior to measuring. Subjects performed a specific warm-up protocol prior to data collection, consisting of two submaximal isometric knee extensions for 5 s at 40, 60, and 80% of MVC force. Each submaximal isometric knee extension was followed by 10-s rest. This was followed by two maximal isometric knee extensions (~5 s) separated by 1 min of rest. During maximal trials, subjects were instructed to increase torque production over a 2-s period and maintain it for 3 s. The protocol for maximal isometric knee flexion followed exactly the same procedures as for extensors. Following knee extensor MVC assessment, subjects received 3 min of rest to avoid fatigue. The highest values from the first two maximal knee extensor and flexor torques for each participant were retained for an analysis. Maximal torque was defined as the maximum value recorded over a period of 0.5 s, after torque had reached a plateau. The corresponding root mean square (RMS) EMG for the selected muscles was quantified during the same 0.5-s period. Average RMS values from the two maximal contractions were retained for normalization of surface EMG obtained during skiing simulations.

Participants then bound their left (outside turn) ski boot into the simulator and were required to maintain the skiing posture by inclining the left “ski” to compensate for the simulated radial force (Figure 3). The inclination for the center of mass in the frontal plane was calculated from the force platform and the load cell measurements and was adjusted to an angle of 25°. For subjects standing on one leg, this inclination produced magnitudes of forces on the outside leg that were comparable with those expected in recreational skiing and were slightly less than those in competitive skiing (Vaverka and Vodickova, 2010; Scheiber et al., 2012; Gilgien et al., 2014). It would have been interesting to simulate greater skier inclination, but pilot measurements with the participants exposed considerable difficulty in maintaining the balance and position of the outer leg on the widest skis. Real-time visual feedback for the magnitude of the inclination of the ski into the turn and for the magnitude of the knee flexion angle was actively used to maintain ski inclination at 25°, as well as for knee flexion at 40° in the first and 60° in the second part of the experiment. After each participant achieved the appropriate posture, a preprogrammed protocol of ski width adjustments was activated. The simulated waist widths of the ski were 0^*^ (W0), 60 (W60), 90 (W90), and 120 (W120) mm; 0^*^ (W0) represented the initial position where the axis of rotation was aligned with the middle of the ski and ski boot in the mediolateral direction. This position served as the starting point of the experiment, creating practically zero torque on the ski around its longitudinal axis, which does not imitate any realistic skiing situation. A motorized tray moved the plate with the ski boot in the aforementioned ski width positions every 8 s in random order. After four adjustments of the ski waist width, there was a 2-min resting period. The procedure was repeated five times for 40° and 60° of knee flexion angles. The calibration procedure for standing upright at 0-mm ski width position was made prior to each skiing simulation.

### Data Processing

Data from the final 2 s of the 8-s measurement periods of each trial and waist width were used for further analysis. For kinematic system measurement, standard Euler's angles in three anatomical planes (Grood and Suntay, 1983) for the simulated outside (left) leg knee joint were acquired in accordance with International Society of Biomechanics recommendations. Relative changes of the knee joint angles with different ski waist widths were calculated by subtracting the data acquired at calibrating upright postures.

The outside torque (*M*) acting on the knee joint was calculated in the frontal plane, whose definition was based on the orientation of the platform (Figure 3) in each instance of time. With the use of GRF and the center of the knee joint, based on the optical marker positions and the standard anatomical knee model, it was possible to calculate the moment arm (*r*) of the GRF acting on the knee joint. Finally, the vector product multiplying *r* and the magnitude of the GRF resulted in the torque value *M*. In addition, from the ratio between the vertical (*F*_v_) and horizontal (*F*_h_) components of GRF, the inclination of the skier's center of mass was calculated as ϕ = arctan(*F*_h_/*F*_v_).

All measured EMG signals were filtered with a band-pass filter (10–500 Hz). An RMS envelope for the EMG signal was calculated using a 300-ms running window. The normalization of the EMG signal was made using the signal at MVC as a reference (detailed description presented above). In this way, the quantity of electrical activity for the selected muscles during the skiing simulation in comparison with the activity at MVC was obtained.

### Statistical Analysis

The data are represented as mean and standard deviation (mean ± SD). Normality of parameter distribution was tested using the Kolmogorov–Smirnov test. One-way repeated-measures ANOVA was conducted for normally distributed data. Bonferroni corrected *post hoc* tests were made to determine the between mean differences in the cases where a significant main effect was seen. In cases where the criteria for repeated-measures ANOVA were not met, the Friedman non-parametric test was used, and paired comparisons were made using Wilcoxon signed-rank tests. In all cases, a *P*-value of 0.05 was accepted as the level of significance.

## Results

### Knee Joint Kinematics

**Figure 4:**
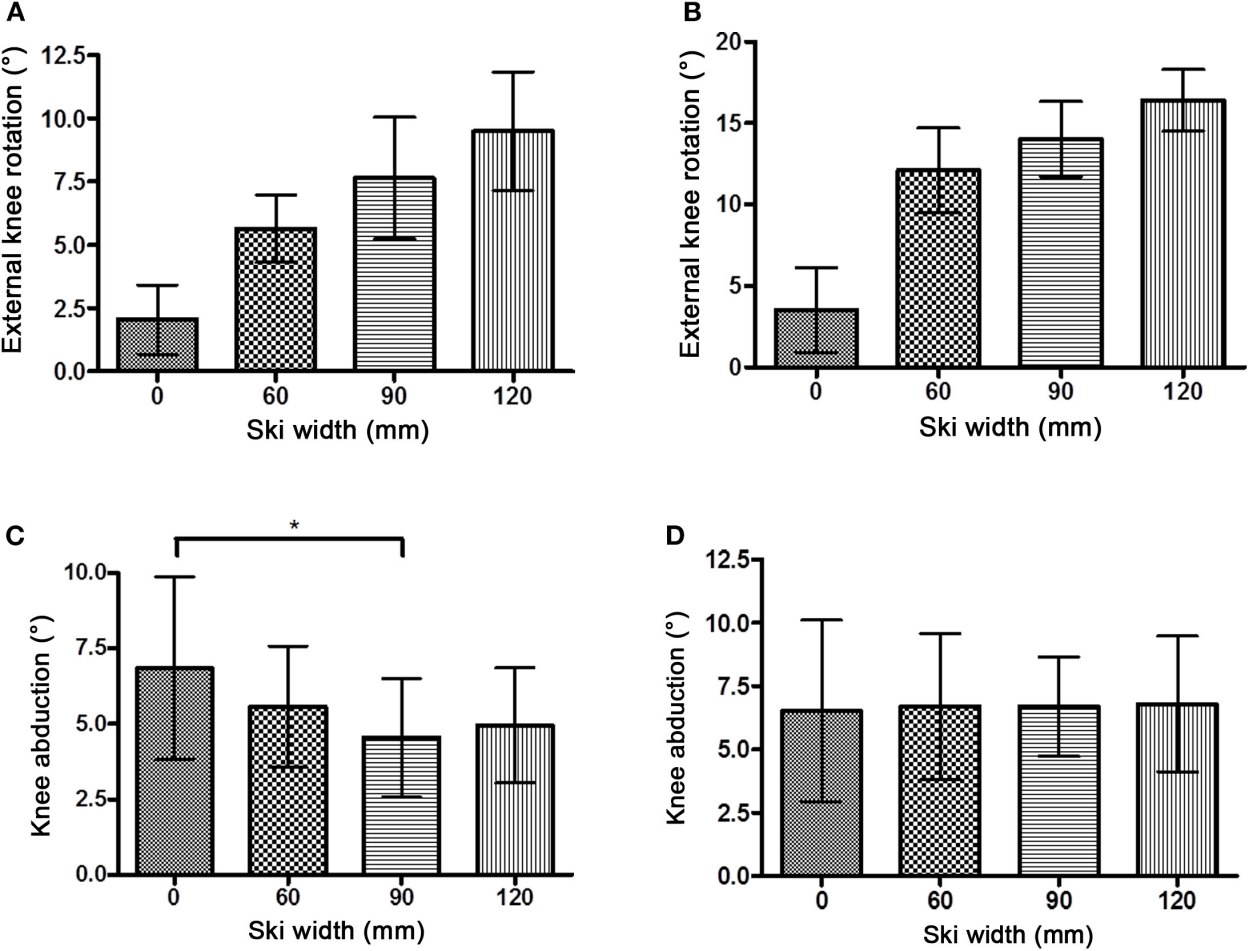
External knee rotation (mean ± SD) with knee flexion 40° **(A)** and 60° **(B)**. A statistical significant increment of the external rotation was observed with each increase in the ski waist width in both knee flexion settings. Knee abduction/valgus (mean ± SD) with knee flexion 40° **(C)** and 60° **(D)**. The statistically significant difference for abduction is marked with “*”.

In all experimental conditions, the knee joint was in external rotation (Figure 4). The Friedman non-parametric test and all pairwise comparisons revealed significant differences between different waist widths for both of the knee flexion experimental conditions, 40° and 60°. The increment of external rotation was the highest for waist widths between W0 and W60 with further increments of external rotation following an almost linear pattern for larger waist width (60 < 90 < 120 mm).

The knee joint was positioned in abducted (valgus) position throughout the experiment (Figure 4). Generally, there were no differences between the magnitude of abduction with different waist widths. The only exception was a statistically significant lower abduction with W90 (4.54 ± 1.96°) compared with W0 (6.84 ± 3.03°, *P* < 0.01) with 40° knee flexion.

### Knee Joint Kinetics

**Figure 5:**
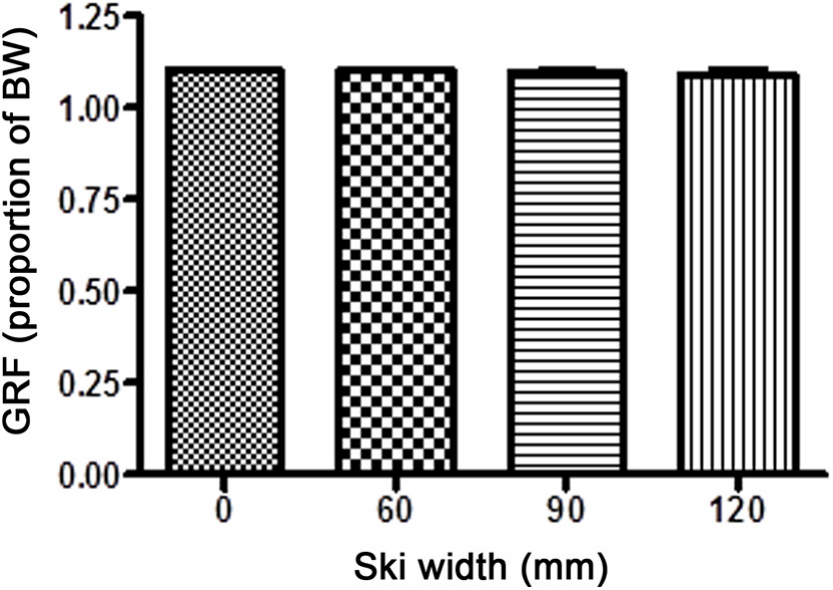
The magnitude of the ground reaction force (GRF; mean ± SD) with different waist widths for both knee flexion settings. There were no statistical differences in GRF between different ski waist widths.

**Figure 6:**
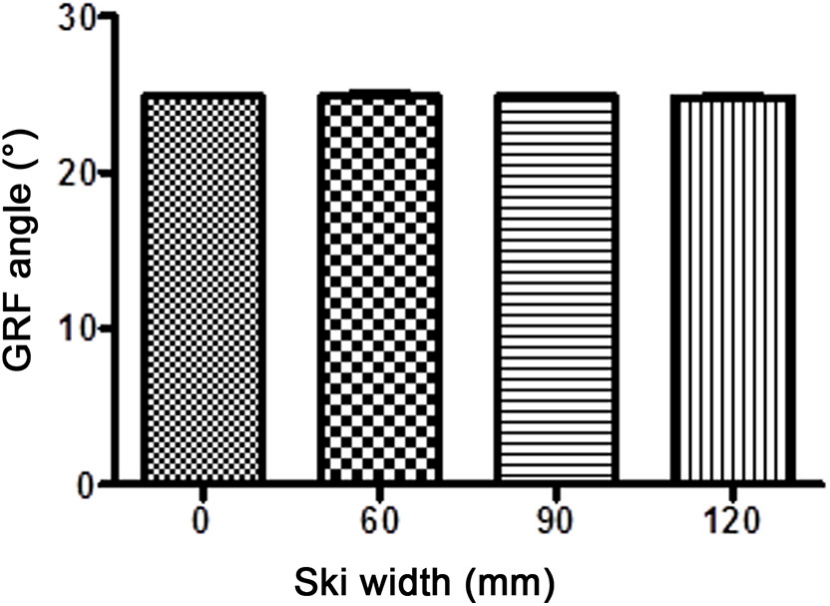
The inclination of the ground reaction force (GRF; mean ± SD) with different waist widths for both knee flexion settings. Throughout the experiment, there were statistically insignificant changes from the preset GRF angle (25° from the vertical line).

**Figure 7:**
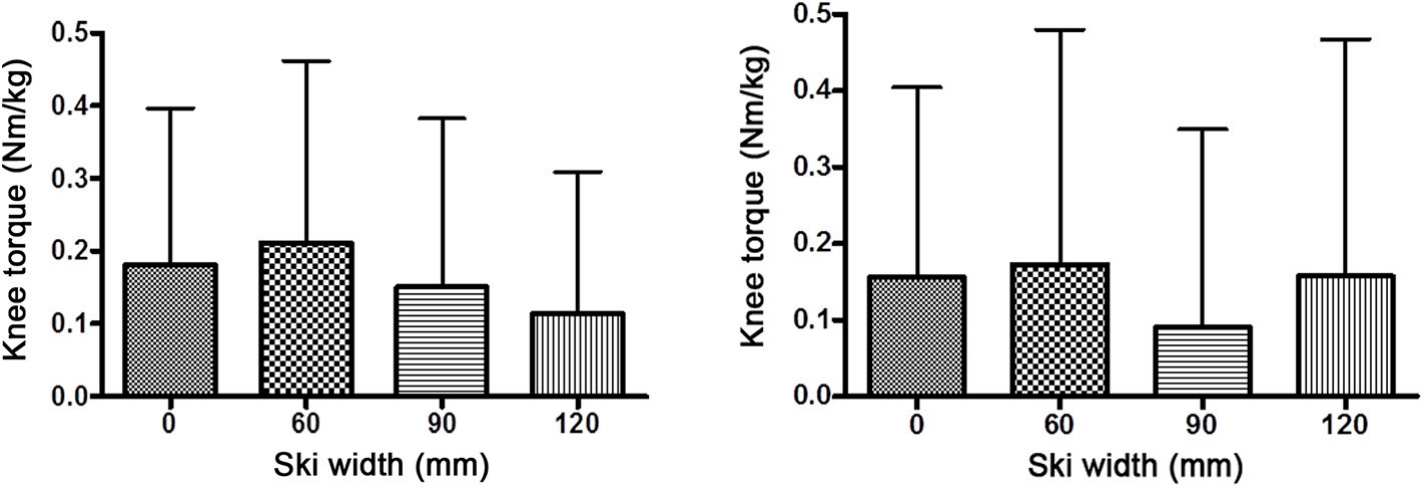
Knee joint torques in the frontal plane (mean ± SD) with knee flexion 40° (left diagram) and 60° (right diagram). There were no statistical differences in knee torques between different ski waist widths.

In all cases, the alignment of GRF in the frontal plane was medial of the knee joint center, with no significant difference between different waist widths. With 40° knee flexion, the distances (*r*) between the knee joint center and GRF were 1.58 ± 1.96 (W0), 1.85 ± 2.13 (W60), 1.38 ± 2.08 (W90), and 1.06 ± 1.86 cm (W120). With 60° knee flexion, the distances were 1.38 ± 2.01 (W0), 1.48 ± 2.48 (W60), 0.84 ± 2.15 (W90), and 1.42 ± 2.54 cm (W120). The magnitude of GRF throughout the experiment remained at the same value as was set at the start (110% body weight, Figure 5). Similarly stands for the angle of GRF (Figure 6). As GRF ran medially to the knee joint center, torque (*M*) acted on the knee in the varus direction. The distribution of *M* for different waist widths is presented in Figure 7. There were no statistical significant differences in magnitude of *M* with different waist widths for either knee joint flexion settings.

### Electromyography

**Figure 8:**
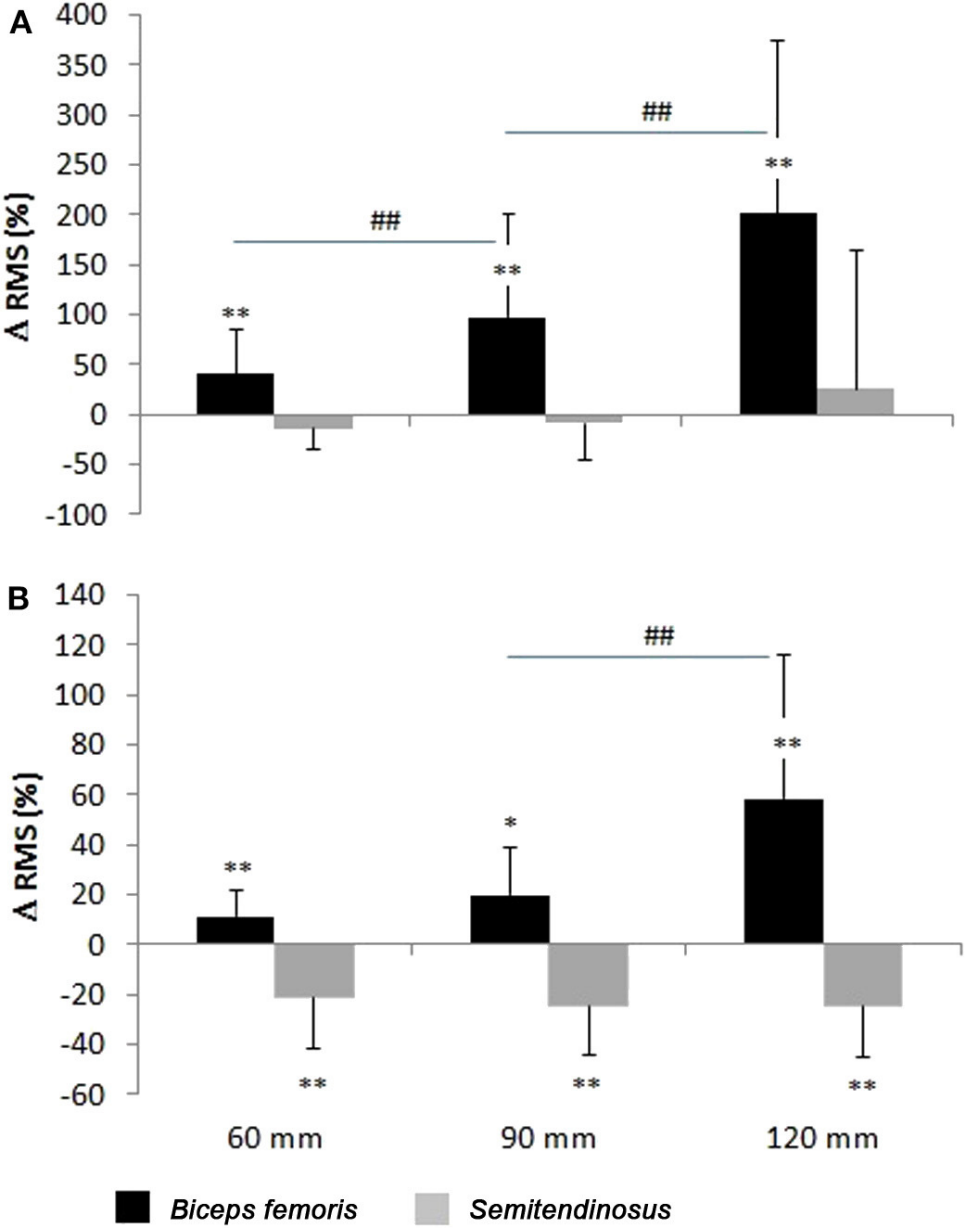
The activation of biceps femoris (BF) and semitendinosus (ST) (mean ± SD) with 40° **(A)** and 60° **(B)** knee flexion. *Depicts statistical significant difference against W(0), and ^#^depicts statistical significant difference with the previous (narrower) waist width.

BF RMS at 40° knee flexion increased throughout the waist width spectrum (Figure 8, Table 1). Likewise, BF RMS at knee flexion 60 increased significantly from 60- to 120-mm waist width. ST RMS remained more or less the same for all waist widths and knee flexion values.

**Table 1:** The RMS values of the surface EMG attained during different ski widths (normalized to maximal RMS attained during MVC).

	**W0**	**W60**	**W90**	**W120**
**40°** **knee flexion**				
BF	12 ± 6%	17 ± 10%^**^	24 ± 16% ^**^^,^^##^	35 ± 25%^**^^,^^##^
ST	8 ± 4%	6 ± 4%	7 ± 4%	8 ± 6%
**60°** **knee flexion**				
BF	16 ± 9%	17 ± 9%^**^	19 ± 10%^*^	24 ± 16%^**^^,^^##^
ST	15 ± 8%	12 ± 8%^**^	11 ± 6%^**^	11 ± 7%^**^

Asterisks indicate significant differences from central position values

(** P < 0.01;

* P < 0.05). Number signs indicate significant differences from narrower position values

(## P < 0.01).

*BF, biceps femoris; ST, semitendinosus; RMS, root mean square; EMG, electromyography; MVC, maximal voluntary contraction*.

## Discussion

The main findings of the study were as follows: (1) the width of the ski has no influence on the external torque acting on knee joint in frontal plane; (2) activation of BF muscle increased by the waist width of the ski in the ski turn; and (3) knee joint kinematics in the transversal plane was influenced by the waist width of the ski during a turn.

### Knee Joint Kinematics

The knee joint of the outer leg in the simulated ski turn was in abducted/valgus and in a relatively externally rotated position, which is in line with previous findings (Yoneyama and Okamoto, 2000). The increase of the external rotation with an increase of the waist width was probably the result of the active adaptation of the skier to reduce the external knee joint torque in the frontal plane. The increase of external rotation was in accordance with the previous field study (Zorko et al., 2015). On the other hand, knee abduction remained the same with any waist width. One might also expect an increment of the abduction with a greater waist width. Greater abduction could possibly bring the knee closer to the GRF vector, that is, avoiding an increase of varus torque. One possible explanation for relatively fixed abduction with any waist width could be that the knee reached the near to end range of motion even with the narrowest skis, which is reported to be 5–10° in the frontal plane for a loose joint position (Grood et al., 1988), and thus, any further increments of abduction were impossible. Instead, a combination of flexion and external rotation in the knee joint with a fixed longitudinal orientation of the ski resulted in the medial movement of the knee joint.

### Knee Joint Kinetics

Torque of the outside knee joint was not influenced by the waist width of the ski. The angle and the magnitude of GRF in the frontal plane remained the same throughout the experiment. The knee joint moment arm in the frontal plane (the orthogonal distance from the GRF vector to the center of the knee joint) also remained the same, despite the point of application of GRF between the narrowest (60 mm) and widest (120 mm) ski width having a displacement of 3 cm. This was only possible with the previously described knee kinematic changes. GRF “pierced” the medial knee joint compartment in the frontal plane within very tight values, 1–2 cm medially to the joint center, which is in accordance with the physiological alignment of GRF by single leg stance (Levine and Bosco, 2007) as well as in accordance with general human limb malalignment adaptive strategies (Mündermann et al., 2005; Levine and Bosco, 2007). Obviously, in possible adverse biomechanical conditions (such as using very wide skis on a hard snow base during turns), the body attempts to retain the unchanged alignment of GRF in relation to knee compartments. This is done by adapting the knee kinematics. Perhaps this is also an attempt to limit the change of the external torque acting on the knee joint.

### Electromyographic Findings

The increment of activation of BF with the waist width increment is in accordance with the changes in the knee external rotation as biceps is also an external rotator of the knee (Besier et al., 2003). The bigger activation at 40° knee flexion compared with 60°, despite a smaller achieved external rotation, is perhaps due to a less feasible rotational knee joint movement at 40° of flexion, where the ligaments are in a tighter position (Platzer, 2004). The magnitude and the ratio of the outer and inner hamstring muscle activations in our study were comparable with those of the average activation of these muscles in a previous field study (Nemeth et al., 1997). Pronounced oscillatory patterns of lower limb muscle activation have been reported in real skiing situations (Panizzolo et al., 2010), so only average values could be compared in the present simulation.

### Methodological Considerations

The crucial concern is whether the laboratory settings sufficiently simulated real alpine skiing turns. The obvious mechanical difference is that the participants had no movement along the sagittal axis. However, taking into account that biomechanical parameters were only in the frontal plane, where at any specific moment in time there is also no movement in real skiing, the performed simulation seems to be suitable. Furthermore, the simulation design allowed some field perturbations to be excluded, as well as provided more superior standardization of the measurement conditions compared with those in a previous study dealing with ski width (Zorko et al., 2015): (i) identical and continuously maintained ski and center of mass inclination angle ensured simulation of the same virtual turning radius for each participant, (ii) by keeping a stationary knee flexion angle, the influence of magnitude of flexion on rotation and abduction angle (Wilson et al., 2000; Lu et al., 2008) and the influence on the lower limb muscle activations were excluded.

At first glance, it may seem that the limitation of the study was also the setting of the skier in a manner that their center of mass did not move during the simulation for different widths of the skis. In other words, the strap giving lateral support to the participant remained unchanged during the sets of movements. One might conclude that all the kinematic and torque changes were merely the consequence of these setting changes. However, it was proven that such small changes of the moving foot relative to the fixed point of the side attachment had a negligible effect on the magnitude (Figure 5) as well as on the alignment of the GRF (Figure 6). Consequently, this setting could not have any influence on the torques of the individual body segments.

Perhaps the single leg stance in our experiment may represent an important difference compared with that in real skiing, where typically both legs are loaded. However, it was shown that the predominant load is normally shifted to the outer ski (Vaverka and Vodickova, 2010). This still might have an impact on the knee torque situation (Klous et al., 2012), which might be different in real skiing. Nevertheless, the loading of the outer leg in our experiment was comparable with the expected average loading of the outer leg during the turn in recreational skiing, if we assume 2:1 force distribution between the outer and inner legs (Vaverka and Vodickova, 2010; Scheiber et al., 2012).

### Summary

The present study has demonstrated that skiers adapt knee joint kinematics with additional external rotation of the tibia against the femur when using wider waist width skis. However, the valgus position of the knee remained independent of the ski waist width. The intention of these kinematical adjustments was probably to maintain alignment of the GRF of the loaded lower limb within close proximity to the joint center, thus minimizing the external torques acting on the joint. Such minimization of the external torques while using very wide waist skis resulted in the knee joint reaching its near end of the range of motion in the frontal and transversal planes combined with higher muscle activation. The probable consequence of using skis with a very large waist width on hard frozen surfaces would be that the knee joint is continuously (during numerous turning) in an externally rotated position and femoral muscles becoming more activated with possible more compression forces acting on joint surfaces. Previous studies (Mündermann et al., 2005; Levine and Bosco, 2007) clarified that the knee joint malalignment is a well-known risk factor for degenerative knee joint conditions. However, whether this type of malalignment and additional muscle activation can lead to long-term knee joint consequences in skiing is yet unclear. Nevertheless, very large waist widths are not permitted in most competitive alpine skiing disciplines, although International Ski Association regulations still have no upper waist width limit in slalom, which has already been pointed out as a potential risk factor (Supej et al., 2017).

### Perspectives

This is the first study to investigate knee joint kinematics, kinetics, and muscle activation in alpine skiing as a function of the ski waist width. Rotation of the tibia against the femur was shown to be progressively influenced by the increment of the waist width of the outer ski during turning. Therefore, increasing waist width potentially escalates uneven joint pressure distribution, while at the same time, this adaptation allows the external torque to remain unchanged. Future epidemiological studies are needed to further elucidate the potential relationship between ski waist width and the damaging effect on the knee joint. Additionally, further improvements in the ski simulations are needed, possibly integrating dynamic movements for imitating turning as well as vibrations, to better simulate real skiing conditions.

## Data Availability Statement

Publicly available datasets were analyzed in this study. This data can be found here: https://plus.si.cobiss.net/opac7/bib/288145920.

## Ethics Statement

This study was carried out in accordance with the recommendations of the responsible Ethics Committee at the University of Ljubljana with written informed consent from all subjects. All subjects gave written informed consent in accordance with the Declaration of Helsinki. The protocol was approved by the responsible Ethics Committee at the University of Ljubljana.

## Author Contributions

MS and MZ designed the study, except for the neurophysiological part, which was designed by KT. MZ, MS, and KT conducted the experiment and collected all the data. BN developed the software for 3D kinematical analysis. ZM and AO developed the skiing simulator and supervised the experimental process. All authors have contributed in the writing of the manuscript, proofread the manuscript, and approved the final version.

### Conflict of Interest

The authors declare that the research was conducted in the absence of any commercial or financial relationships that could be construed as a potential conflict of interest.
